# The neural basis of non-verbal communication—enhanced processing of perceived give-me gestures in 9-month-old girls

**DOI:** 10.3389/fpsyg.2015.00059

**Published:** 2015-02-06

**Authors:** Marta Bakker, Katharina Kaduk, Claudia Elsner, Joshua Juvrud, Gustaf Gredebäck

**Affiliations:** ^1^Uppsala Child and Baby Lab, Department of Psychology, Uppsala University, Uppsala, Sweden; ^2^Department of Psychology, Fylde College, Lancaster University, Lancaster, UK

**Keywords:** give-me gesture, ERP, P400, sex differences, non-verbal communication, social perception, infancy

## Abstract

This study investigated the neural basis of non-verbal communication. Event-related potentials were recorded while 29 nine-month-old infants were presented with a give-me gesture (experimental condition) and the same hand shape but rotated 90°, resulting in a non-communicative hand configuration (control condition). We found different responses in amplitude between the two conditions, captured in the P400 ERP component. Moreover, the size of this effect was modulated by participants’ sex, with girls generally demonstrating a larger relative difference between the two conditions than boys.

## INTRODUCTION

Gestures may be used as social tools for expressing one’s own feelings and thoughts, cooperating with others, and drawing others’ attention to objects and events (Tomasello et al., 2007; Carpendale and Carpendale, 2010). In early childhood gestures may be expressed in grimaces and smiles (Caselli, 1990) and are later exhibited with fingers, hands, and arms (Crais et al., 2004). By the end of the first year of life, gestures such as giving (Mundy et al., 1986; Caselli, 1990) or pointing (Bates et al., 1975; Tomasello, 2008) become meaningful for expressing goals and communicating with others.

Research exploring the development of the pointing gesture is quite prevalent (e.g., Butterworth, 2003; Camaioni et al., 2004; von Hofsten et al., 2005; Liszkowski et al., 2006; Tomasello et al., 2007; Daum et al., 2013). In contrast, the give-me gesture (a face-up palm directed toward the observer; Mundy et al., 1986) has received little attention. We believe that the give-me gesture warrants more interest from the scientific community considering its communicative importance in serving multiple functions, such as referring to a specific object, expressing a request and communicating an action goal (Shwe and Markman, 1997).

From a behavioral perspective, we know that children begin to give and request objects to and from others at around 9- to 12-month of age (Bates et al., 1975; Masur, 1983; Carpenter et al., 1998; Crais et al., 2004). Recent eye tracking studies show that infants are sensitive to the communicative properties of the give-me gesture by 12-month of age (Elsner et al., 2014). In this study, infants observed a give-and-take interaction between two individuals. At the beginning of each trial the receiving hand formed either a give-me gesture or an inverted hand shape (hand shaped as a give-me gesture but presented upside-down). Subsequently, the passing hand (hand from another individual) that was located on the opposite side of the screen transferred the ball to a receiving hand. The authors assessed differences in latency of goal-directed gaze shifts from the hand transporting the ball to the receiving hand. The results revealed that infants shifted their gaze significantly earlier toward the goal, the receiving hand, if it was shaped as a give-me gesture in comparison to an inverted hand shape. Additional control conditions ruled out that the effect was based on affordance, e.g., a simple match between the ball and the receiving hand, or attentional differences (Elsner et al., 2014). Jointly, the results indicate that infants are sensitive to the communicative intent of a hand shaped in a give-me gesture. Another eye tracking study demonstrated that 14-month-old infants have a clear expectation of adequate responses to the give-me gesture. That is, when observing an interaction between two people, infants anticipate that an object will be passed to another person when the give-me gesture request is presented, suggesting again that infants at this age can recognize the communicative intent of the gesture (Thorgrimsson et al., 2014). Interestingly, perception of give-me gestures may be different for typically developing children than children with autism spectrum diagnosis (ASD). In a recent study 5- to 6-year old children with ASD were found to look differently at social interactions incorporating give-me gestures differentially than typically developing children (Falck-Ytter et al., 2013). This may suggest that children with this clinical diagnosis might be less able to read the meaning of the give-me gesture or that they are less interested in the people’s reactions that are confronted with give-me gestures (Falck-Ytter et al., 2013).

Motivated by eye tracking studies that highlight the importance of the give-me gesture in goal understanding and encoding social interaction during development (Falck-Ytter et al., 2013; Elsner et al., 2014; Thorgrimsson et al., 2014), as well as a desire to learn more about the neural mechanisms that are involved in processing of give-me gestures, the current study investigated the neural activation that is evoked when observing give-me gestures. To our knowledge, only two studies have investigated the neural correlates of gesture perception early in development. The first study investigated the neurodevelopment of pointing perception (Gredebäck et al., 2010), whereas the second the perception of grasping gestures (Bakker et al., 2014). In those studies, the authors reported the ERP component P400 to be sensitive to the congruency of pointing or grasping, revealing higher mean amplitudes for the congruent (gestures directed toward an object) compared to the incongruent condition (gestures directed away from the object). Here, we aim to explore if the same ERP component generalizes over communicative settings, from hand configurations directed toward objects (pointing; Gredebäck et al., 2010, and grasping; Bakker et al., 2014) to more socially oriented gestures, in this case the give-me gesture directed toward the infant. If the same underlying neural processes are involved in processing of a large array of gestures, than we would expect larger amplitudes of the P400 for the give-me gesture than a hand configuration that is perceptually very similar but has no communicative intent (from here labeled as non-communicative hand configuration).

In addition, we aim to investigate the relation between infants’ neural response to the give-me gesture and infants’ own ability to respond to the same gesture on a behavioral level. Prior work has demonstrated that infants process both pointing (Gredebäck et al., 2010) and grasping gestures (Bakker et al., 2014) by 9 months of age. At the same age, infants also start to engage in producing give-me gestures (Bates et al., 1975; Masur, 1983; Carpenter et al., 1998; Crais et al., 2004). Based on the revealed correspondence between infants’ neural potentials and behavior in prior EEG studies (i.e., Bakker et al., 2014), the current study targets both 9-month-old infants’ neural correlates of the give-me gesture and their behavioral responses to give-me requests (Responding to Behavioral Request procedure from the Early Social Communication Scales; Mundy et al., 2003). We expect that behavioral responses to the give-me gesture will correspond with P400 amplitudes. That is, relative amplitudes (give-me gesture vs. non-communicative hand configuration) should be higher in infants that are proficient in responding behaviorally to the give-me gesture.

Further analyses in this study explored individual differences in gesture perception with respect to infants’ sex. Based on prior studies revealing that girls are ahead of boys in the onset of gesture and language production (Butterworth and Morissette, 1996; Özçalşkan and Goldin-Meadow, 2010), it is possible that girls are more proficient in discriminating between the give-me gesture and the non-communicative hand configuration than boys. If we find such an effect we would expect an interaction effect between sex and condition. That is, both boys and girls should be able to differentiate between the two conditions, but we would expect the effect to be bigger in girls than boys.

In summary, the current study has three aims: to investigate the give-me gesture perception on a neural level, to investigate infants’ behavioral response to the give-me gesture and to investigate the presence of sex differences in social perception mechanisms.

## MATERIALS AND METHODS

### PARTICIPANTS

The final sample consisted of twenty-nine 9-month-olds (15 girls, mean age 8 months and 28 days, SD = 6 days). An additional 30 infants (16 girls) participated but were excluded due to fussiness (less than 10 artifact-free trials, *n* = 25) or technical problems (*n* = 5). Parents completed informed consent prior to participation and received a gift voucher of approximately 10° for participating. The study was conducted in accordance with the standards specified in the 1964 Declaration of Helsinki and approved by the local ethics committee.

### EEG STIMULI

**FIGURE 1:**
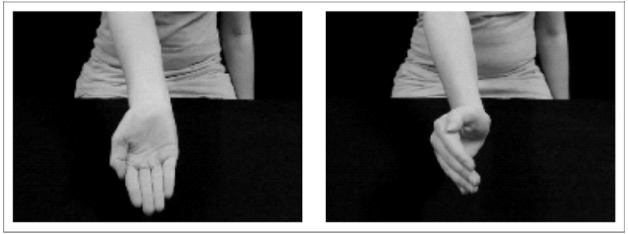
**Stimulus for the give-me gesture condition on the left and a control hand on the right**.

The give-me gesture (experimental condition) and the non-communicative hand configuration (control condition) were presented to the infants. In both conditions the stimulus included a hand (palm facing upward in the experimental condition and the same hand rotated 90° in the control condition). Stimuli were presented at random (with the constrains of maximum three repetitions of the same stimulus) and presented in the middle of a gray background for 1000 ms. Between each experimental stimulus; a fixation cross was presented for 100–300 ms (see Figure 1). Infants viewed the stimuli (20.7 × 16.5 visual degrees) on a 17-inch computer monitor at a viewing distance of 60 cm. The size of the hand was 5 horizontal and 16 vertical visual degrees. The stimuli were presented using the E-Prime 2.0, E-Studio software (Psychology Software Tools, Inc., Pittsburgh, PA, USA).

### BEHAVIORAL TASK

Parents were asked if they have observed their child producing or responding to the give-me gesture outside of the laboratory. Subsequently, a researcher assessed infant’s behavioral response to the give-me gesture using the Responding to Behavioral Request procedure from the ESCS (Mundy et al., 2003). The experimenter first familiarized the infant with three rubber toys (5 × 5 cm) and then placed the toys in front of the infant and waited (3 s) for the infant to give the toy back spontaneously. If the infant did not pass a toy, the experimenter verbally requested the toys with the phrase: “give it to me.” If after 3 s the infant did not respond to the verbal request, the experimenter used a combination of verbal request together with a non-verbal give-me gesture. The experimenter’s gesture stopped within reach of the infant. The infant’s behavior was video recorded and later assessed for the frequency of appropriate responses, that is, the number of times the child gave a toy to the experimenter at the request (verbal or verbal in combination with the give-me gesture). The total duration of this grasping test did not exceed 5 min.

### PROCEDURE

During the lab visit, we first recorded infants’ neural responses to the give-me gesture, followed by a behavioral task that measured the ability to respond to the give-me gesture. During the EEG recording, infants sat on their parent’s lap approximately 60 cm from the stimulus monitor. The experimenter sat at a control computer separated from the parent and infant by a curtain and monitored the infant’s behavior via a live camera. The researcher paused the experiment if the infant became inattentive and fussy. The stimulus monitor remained black for the duration of the pause. The experimenter terminated the study when the infant was no longer interested in the stimuli. After the EEG recording the parent and infant were given an approximate 5 min break before proceeding with the behavioral response task. This paper reports data from an ongoing longitudinal project looking at the neural correlates of social cognition and later language development.

### EEG RECORDING AND ANALYSIS

**FIGURE 2:**
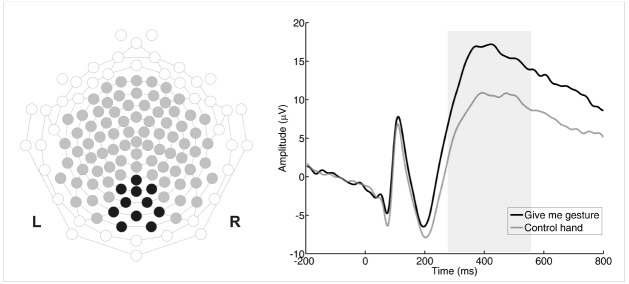
**Grand-average of ERP of the posterior area (channels of interest are marked in black).** Black line represents the give- me gesture condition and grey line the control hand.

We used 128-channel HydroCel Geodesic Sensor Nets to record infants’ EEG. The recorded signal (250 Hz, vertex referenced) was amplified by an EGI Net Amps 300 amplifier (Electric Geodesic, Eugene, OR) and stored for off-line analysis. The EEG signal was digitally filtered (0.3–30 Hz) and segmented from 200 ms prior to the appearance of the hand to 1000 ms after the onset of the stimulus. Off-line inspection of video recordings ensured that only trials in which infants paid attention were further processed. The data was manually edited for artifacts (standard procedure for infant ERP studies, see Hoehl and Wahl, 2012). Trials with excessive noise levels (mostly due to movement artifacts) were rejected. Channels with moderate noise levels were reconstructed from an interpolation of surrounding electrodes. All included trials contained no more than 10% interpolated channels. The whole recording session did not exceed 10 min. The inclusion criterion for the final analysis was at least 10 artifact free trials per condition (standard inclusion criterion for infants ERP studies, see DeBoer et al., 2007; Stets et al., 2012). On average, an infant saw 90 trials across both conditions, with 44 trials for the give-me gesture condition and 46 for the control hand. After visual data inspection and manual data editing, a mean of 15 artifact free trials remained (range: 10–31) for the give-me gesture condition and a mean of 17 trails (range: 10–32) for the control hand. Finally, we baseline corrected and averaged all artifact free trials, as well as re-referenced to the average in order to create individual averages for each participant, as well as calculated grand averages from individual averages. Based on the visual inspection of the individual averages and grand average we selected 11 channels in the posterior area (62, 67, 70 71, 72, 74, 75, 76, 77, 82, 83) for statistical analyses. We captured three components in the ERP wave morphology after the stimulus onset, and performed the analysis in the following three time windows (see Figure 2): P1 (80–140 ms), N200 (150–250 ms) and P400 (300–600 ms). We conducted analyses of variance (ANOVAs) to compare the mean amplitudes between conditions (the give-me gesture and control) in all ERP components (P1, N200, P400) and to assess the effect of sex on ERP amplitude differences, respectively.

## RESULTS

### ERPs

**FIGURE 3:**
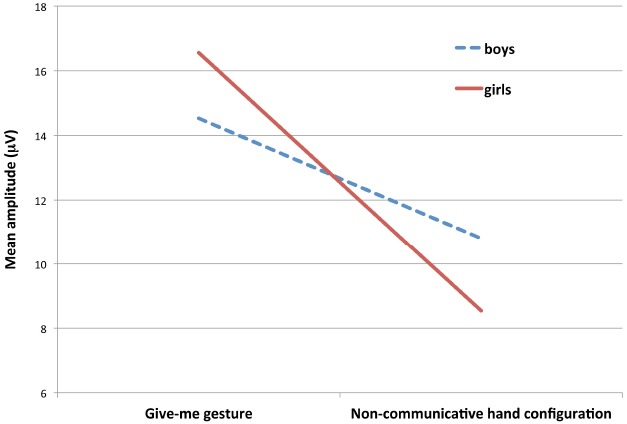
**Mean amplitude P400 separately for each condition and sex.** Red and blue-dashed lines illustrate the interaction between Condition and Sex.

Our first ERP analysis focused on the component of interest, the P400. In order to test the possible difference between the conditions as well as the effect of sex on the modulation of the P400 amplitude, we conducted a 2(sex) × 2(condition) mixed repeated measures ANOVA. Results revealed a main effect of condition *F*(1,27) = 40.12, *p* < 0.001, η^2^ = 0.598, with a mean amplitude of 15 μV (SD = 6 μV) in response to the give-me gesture and 9 μV (SD = 7 μV) in response to seeing the non-communicative hand configuration. Overall, 26 out of 29 infants demonstrated larger amplitudes for the give-me gesture compared to the non-communicative hand configuration. Additionally, there was a significant interaction between Condition and Sex [*F*(1,27) = 5.384, *p* = 0.028, η^2^ = 0.166; see Figure 3]. To inspect the condition by sex interaction, we performed planned comparison paired-samples *t*-tests (separately for each sex). Results revealed significant differences between conditions, both for girls [*t*(27) = 4.750, *p* < 0.001] as well as for boys [*t*(27) = 4.360, *p* < 0.001] with more positive mean amplitudes for the give-me gesture. As both boys and girls displayed a significant difference in their response to the two gestures, and as the direction of the difference was similar, it is possible that the interaction between Sex and Condition stems from differences in the size of the effect. To test this prediction, we further examined the difference between the sexes in their conditional amplitude difference scores. We performed an independent-samples *t*-test with the amplitude difference as a dependent variable and sex as a grouping variable. The analysis revealed a significant amplitude difference between the sexes [*t*(27) = 2.320, *p* = 0.028], This clearly shows that the interaction is driven by the size of the difference between the conditions that is larger for girls (girls: *M* = 8 μV, SD = 6 μV; boys: *M* = 10 μV, SD = 8 μV).

To ensure that the effect between conditions as well as the interaction between Condition and Sex is specific to the P400 we performed a follow-up analysis for two other components visible in the ERP wave morphology, i.e., P1 and N200. We performed a 2 × 2 mixed repeated measures ANOVAs with Condition as a within-subject factor and Sex as between-subject factor on the mean amplitudes of the P1 and N200. The analysis for the P1 component revealed no significant effects, neither for difference between conditions [*F*(1,27) = 2.297, *p* = 0.141, η^2^ = 0.078] nor an interaction between Condition and Sex [*F*(1,27) = 2.149, *p* = 0.154, η^2^ = 0.074]. The analysis for the N200 also failed to show significance, neither for differences between the conditions [*F*(1,27) = 2.808, *p* = 0.105, η^2^ = 0.094] nor for an interaction [*F*(1,27) = 0.077, *p* = 0.783, η^2^ = 0.003].

### BEHAVIORAL TASK

On a behavioral level, none of the infants responded to the give-me gesture request as determinated by the ESCS scale. Four infants responded by moving the hand with the object to the experimenter but did not release it. Two infants moved the hand away from the experimenter when seeing the request. None of the caregivers reported that their infant was able to produce or respond to the give-me gesture outside the laboratory. Therefore, no statistical analysis was performed.

## DISCUSSION

This study investigated infants’ neural correlates to the perception of the give-me gesture, a non-verbal communication. As predicted, we found that infants’ P400 component increased in amplitude when infants were presented with the give-me gesture compared to a non-communicative hand configuration. This difference was significant despite the fact that most of the infants did not demonstrate an overt sensitivity to the give-me gesture (as measured with ESCS).

The current study is the first to demonstrate neural correlates to give-me gestures in 9-month-old infants. Furthermore, we demonstrate that the neural basis of non-verbal communication, as indexed by the sensitivity to the give-me gesture, develops before overt responses to other people’s give-me gestures. It is possible that our results capture an early neural sensitivity that is a functional prerequisite of later overt behavior. As all intentional behavior must have its neural underpinnings, it is possible that the neural support networks must first be in place in order for overt behavior to emerge. For a more immediate connection between referential gesture communication and infants’ own motor abilities in the case of grasping, see Bakker et al. (2014). Finally, as predicted, we demonstrate sex differences in the neural responses to the give-me gesture, with larger amplitude difference between conditions in girls than boys.

### P400—NEURAL CORRELATE OF THE GIVE-ME GESTURE

In the current study we found that the give-me gesture elicits larger P400 amplitude than the non-communicative hand configuration in 9-month-old infants. This effect is highly similar to the neural response elicited while observing goal-directed pointing (Gredebäck et al., 2010) and grasping (Bakker et al., 2014). In those studies, the amplitude of the P400 was larger for typical and functional referential cues (i.e., give-me gesture, congruent pointing, congruent reaching) than for the control stimuli that were less communicative or functional. Here, we demonstrate similar differences in the amplitude of P400 for gestures directed toward the infant. Together, these findings demonstrate that the P400 indexes a wide range of social gestures, comprising both gestures directed toward objects and those directed toward the observing infant.

In contrast to prior studies examining neural correlates in relation to behavioral response of pointing and grasping, we did not find a relation between P400 ERP to give-me gesture and infants’ behavioral response to the same gesture. In the prior study on grasping perception (Bakker et al., 2014), 5–6 months old infants’ own experience with grasping was closely connected to their ability to encode the relation between the presented object and the grasping hand. More specifically, a difference in the P400 between conditions (hand directed toward or away from the object location) was only evident in infants that were able to perform functional grasping. In the current study, however, infants that did not show a behavioral response to the give-me gesture showed a clear sensitivity in evoked ERPs to this gesture. It is possible that the neural correlates of basic action perception and action production develop simultaneously for actions that emerge early during infancy (like grasping). However, gestures like the give-me gesture are more complex and a proper behavioral response may require more understanding of properties of the gesture and turn-taking in social interactions.

More research is required to further examine the developmental trajectories of the perception and production of give-me gestures. Longitudinal designs investigating the relation between functional and behavioral aspects of give-me gesture perception could provide new perspectives on the development of non-verbal communication and infants’ understanding of cooperative actions. Additionally, it would be valuable to gain an understanding on whether the give-me gesture relates to other referential gestures and referential cues on both a behavioral and neural level. The combination of neural and behavioral measures would expand our knowledge about infants’ early communicative development, which so far has been limited to pointing, even though infants’ gestural repertoire is more extensive.

### INDIVIDUAL DIFFERENCES IN PERCEPTION OF GIVE-ME GESTURES

In the current study we found a significantly larger difference between conditions in P400 amplitudes for girls than for boys. This difference is interpreted as an indication that girls might be more sensitive to discriminating give-me gestures from other non-communicative hand configurations. To our knowledge there are no EEG studies that have reported sex differences in social perception in infancy. Some sex differences have, however, been observed in infant studies that used behavioral measures. For instance, differences between boys and girls have been demonstrated in the frequency of eye contact between the child and the mother, with girls making more eye contact than boys (Lutchmaya et al., 2002). It has also been suggested that infant girls may be more attracted to social stimuli than boys, for example when being presented with faces (Lutchmaya and Baron-Cohen, 2002) or abstract geometric shapes chasing each other (Frankenhuis et al., 2013) and faces (Lutchmaya and Baron-Cohen, 2002). In a meta-analytic review of sex differences in facial expression processing in infancy, McClure (2000) reported that females outperformed males in interpreting facial expressions and other non-verbal cues. These advantages for females are visible both in infancy as well as in adulthood. A recent study that inspected brain activation during observation of biological motion revealed a difference between adult female and male participants, with females showing greater activation in brain regions that are involved in social perception (Anderson et al., 2013). The authors also found a similar trend in children (Anderson et al., 2013). Based on these findings it is likely that the sex differences found in the present study would replicate across a larger range of social perception studies examining neural processes targeting social stimuli. Furthermore, we speculate that the results from this study capture possible sex differences in processing of non-verbal cues. This is in line with previous research that reported females being more accurate in decoding non-verbal cues (Hall, 1978), joint attention and communicative skills (Olafsen et al., 2006). Additionally, Özçalşkan and Goldin-Meadow (2010) found that the onset of gesture and sentence production emerges later in boys than girls. In this context it is important to note that the current study captures sex differences in response to non-verbal social cues at an extremely early age, before the actual onset of gesture and speech production.

Taken together, we believe, that higher average P400 amplitude found in this study was generated by infants’ encoding of more communicative intent in the give-me gestures in comparison to non-communicative hand configuration. It is worth mentioning that again that no differences were found in ERP components (P1) that often index pure visual differences in stimuli. Additionally, prior work has also conducted several controls that rule out affordance and visual attention as alternative explanations (Elsner et al., 2014). As a whole, the P400 literature suggests that infants from an early age perceive functional and goal-directed manual actions and gestures in a similar manner. These processes operate both during observation of manual gestures directed toward objects as well as toward the observing infant. All of these events result in larger amplitude modulation in comparison to non-goal directed or non-communicative hand configurations.

In conclusion, the current study is the first to examine neural underpinnings of the give-me gesture. Our findings contribute to the understanding of the P400 neural component suggesting an involvement in encoding social interactions and non-verbal communication. More specifically our study demonstrates that the P400 is sensitive to observation of the give-me gesture with 9-month-old girls demonstrating a larger difference between conditions than 9-month-old boys.

### Conflict of Interest Statement

The authors declare that the research was conducted in the absence of any commercial or financial relationships that could be construed as a potential conflict of interest.
